# Inhibition of Mitochondrial Complex-1 Prevents the Downregulation of NKCC2 and ENaCα in Obstructive Kidney Disease

**DOI:** 10.1038/srep12480

**Published:** 2015-07-24

**Authors:** Yue Zhang, Ying Sun, Guixia Ding, Songming Huang, Aihua Zhang, Zhanjun Jia

**Affiliations:** 1 Department of Nephrology, Nanjing Children’s Hospital, Affiliated with Nanjing Medical University, Nanjing 210008, China; 2 Institute of Pediatrics, Nanjing Medical University, Nanjing, China; 3 Nanjing Key Laboratory of Pediatrics, Nanjing Children Hospital, Affiliated with Nanjing Medical University, Nanjing 210008, China

## Abstract

Ureteral obstruction with subsequent hydronephrosis is a common clinical complication. Downregulation of renal sodium transporters in obstructed kidneys could contribute to impaired urinary concentrating capability and salt waste following the release of a ureteral obstruction. The current study was undertaken to investigate the role of mitochondrial complex-1 inhibition in modulating sodium transporters in obstructive kidney disease. Following unilateral ureteral obstruction (UUO) for 7 days, a global reduction of sodium transporters, including NHE3, α-Na-K-ATPase, NCC, NKCC2, p-NKCC2, ENaCα, and ENaCγ, was observed, as determined via qRT-PCR and/or Western blotting. Interestingly, inhibition of mitochondrial complex-1 by rotenone markedly reversed the downregulation of NKCC2, p-NKCC2, and ENaCα. In contrast, other sodium transporters were not affected by rotenone. To study the potential mechanisms involved in mediating the effects of rotenone on sodium transporters, we examined a number of known sodium modulators, including PGE2, ET1, Ang II, natriuretic peptides (ANP, BNP, and CNP), and nitric oxide synthases (iNOS, nNOS, and eNOS). Importantly, among these modulators, only BNP and iNOS were significantly reduced by rotenone treatment. Collectively, these findings demonstrated a substantial role of mitochondrial dysfunction in mediating the downregulation of NKCC2 and ENaCα in obstructive kidney disease, possibly via iNOS-derived nitric oxide and BNP.

Obstructive kidney disease is a common clinical complication^1^,^2^,^3^,^4^. In children, kidney obstruction is usually caused by congenital abnormalities of the kidneys and urinary tract^5^,^6^,^7^. In adults, the most common causes are stones, prostate enlargement, and tumors of the urinary tract^1^,^2^,^3^. Generally, kidney dysfunction is reversible when the obstruction is released within a short time period. However, long-term kidney obstruction leads to kidney fibrosis and permanent loss of renal function. An established phenomenon in obstructive nephropathy is the downregulation of sodium transporters in the obstructive kidney, which could contribute to urinary concentrating defects following the release of the kidney obstruction. Both cyclooxygenase (COX)-2-derived prostaglandin (PG) E2 and angiotensin (Ang) II have been reported to be attributable to the reduction of sodium transporters in the obstructed kidney^8^,^9^. However, more intensive study will be necessary to reveal the pathogenesis of this phenomenon in detail.

Mitochondrial abnormality has been reported in obstructive nephropathy^10^,^11^,^12^,^13^. Mitochondrial dysfunction results in ATP depletion, reactive oxygen species (ROS) overproduction, and the release of proapoptotic factors such as cytochrome C and mitochondrial DNA. These abnormal responses could play important roles in the pathogenesis of many diseases. In agreement with this concept, our group reported that inhibition of mitochondrial complex-I attenuated obstructive kidney injury, possibly via inhibition of oxidative stress, inflammation and fibrosis^13^. A global downregulation of sodium transporters in the obstructed kidney is thought to be one of key factors leading to impairment of the renal concentrating capability^8^,^9^. However, our understanding of the mechanisms leading to such abnormalities is still incomplete.

In the present study, employing the mitochondrial complex-1 inhibitor rotenone, we investigated 1) whether inhibition of mitochondrial complex-I could affect the downregulation of sodium transporters in the obstructed kidney; and 2) whether some known diuretic factors were affected by rotenone treatment and potentially contributed to the effect of rotenone on regulating sodium transporters.

## Results

### Effects of mitochondrial complex-I inhibition on the mRNA expression of sodium transporters in obstructed kidneys

**Figure 1:**
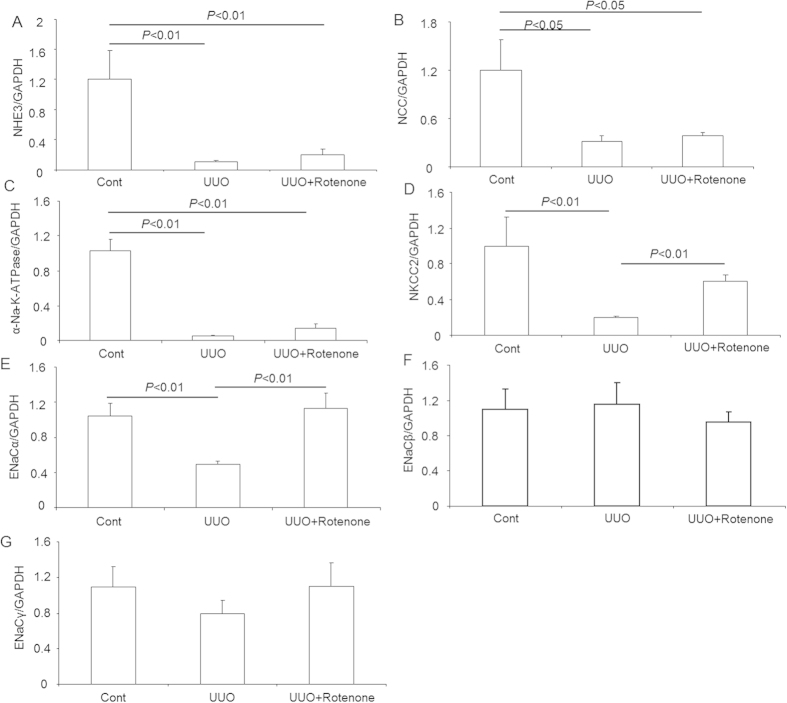
mRNA expression of sodium transporters in obstructed kidneys following rotenone treatment. (**A**) qRT-PCR analysis of NHE3. (**B**) qRT-PCR analysis of NCC. (**C**) qRT-PCR analysis of α-Na-K-ATPase. (**D**) qRT-PCR analysis of NKCC2. (**E**) qRT-PCR analysis of ENaCα. (**F**) qRT-PCR analysis of ENaCβ. (**G**) qRT-PCR analysis of ENaCγ. The presented data are means ± SE. N = 5 in each group.

To study the role of rotenone treatment in the regulation of sodium transporters in obstructive kidney disease, we examined the mRNA expression of sodium transporters including NHE3, α-Na-K-ATPase, NCC, NKCC2, and three ENaC subunits (α, β, and γ) via qRT-PCR. The data showed that NHE3, α-Na-K-ATPase, and NCC were markedly downregulated in obstructed kidneys, which was not affected by rotenone administration (Fig. 1A–C). In contrast, a marked reduction of NKCC2 mRNA expression was partially, but significantly reversed by rotenone treatment (Fig. 1D). For the three ENaC subunits, the downregulation of ENaCα in obstructed kidneys was completely inhibited by rotenone (Fig. 1E). However, the mRNA levels of ENaCβ and ENaCγ were not altered by ureteral obstruction or rotenone treatment (Fig. 1F,G).

### Effects of mitochondrial complex-I inhibition on the protein expression of NKCC2 and the phosphorylation of NKCC2 in obstructed kidneys

**Figure 2:**
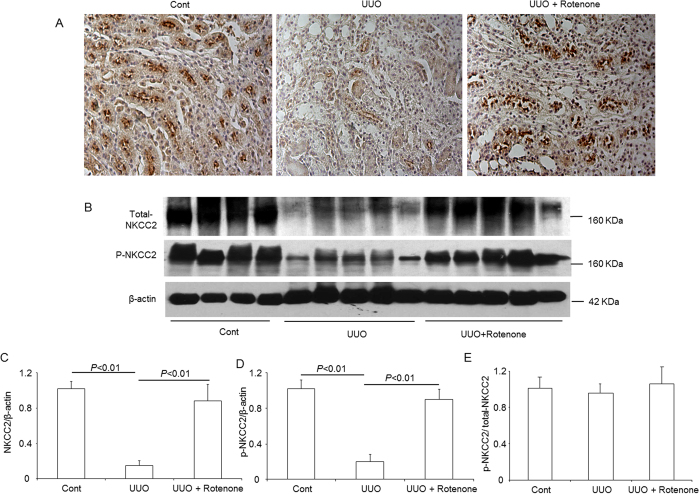
Protein expression of total NKCC2 and p-NKCC2 in obstructed kidneys following rotenone treatment. (**A**) Immunohistochemistry of NKCC2. (**B**) Western blot analysis of NKCC2 and p-NKCC2. (**C**) Densitometric analysis of NKCC2. (**D**) Densitometric analysis of p-NKCC2 normalized by β-actin. (**E**) Densitometric analysis of p-NKCC2 normalized by total NKCC2. The presented data are means ± SE. N = 4–5 in each group.

The immunohistochemistry results showed that the robust downregulation of NKCC2 protein expression in the obstructed kidney was entirely prohibited by rotenone treatment (Fig. 2A). Similarly, Western blotting revealed a striking reduction of NKCC2 levels and complete restoration upon rotenone administration (Fig. 2B,C). To investigate the status of NKCC2 phosphorylation, we further examined the levels of phosphorylated NKCC2 through Western blotting and observed a similar regulatory pattern to total NKCC2 (Fig. 2B,D). However, the ratio of p-NKCC2 to total NKCC2 was not affected by kidney obstruction or rotenone treatment (Fig. 2E), indicating that the change of p-NKCC2 was resulted from the alteration of total NKCC2. These results demonstrated a potent role of mitochondrial complex-I inhibition in inhibiting the downregulation of NKCC2 in the thick ascending limbs of obstructed kidneys.

### Effects of mitochondrial complex-I inhibition on the protein expression of ENaC subunits in obstructed kidneys

**Figure 3:**
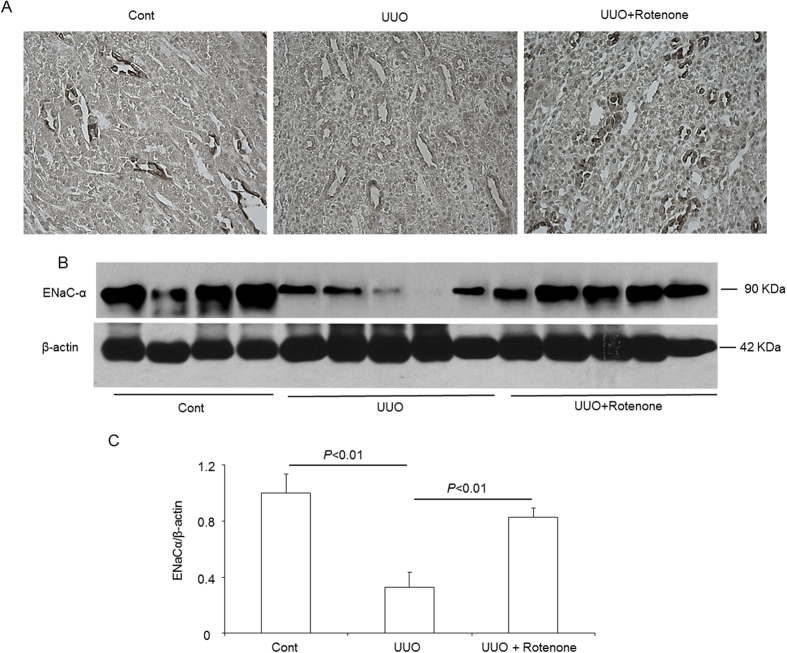
Protein expression of ENaCα in obstructed kidneys following rotenone treatment. (**A**) Immunohistochemistry of ENaCα. (**B**) Western blot analysis of ENaCα. (**C**) Densitometric analysis of ENaCα. The presented data are means ± SE. N = 4–5 in each group.

**Figure 4:**
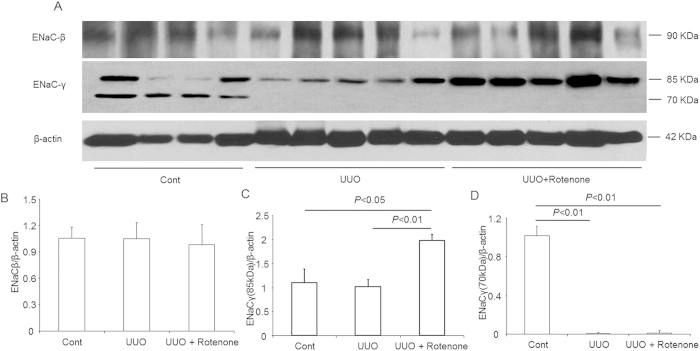
Protein expression of ENaCβ and ENaCγ in obstructed kidneys following rotenone treatment. (**A**) Western blot analysis of ENaCβ and ENaCγ. (**B**) Densitometric analysis of ENaCβ. (**C**) Densitometric analysis of ENaCγ (85 kDa). (**D**) Densitometric analysis of ENaCγ (70 kDa). The presented data are means ± SE. N = 4–5 in each group.

**Figure 5:**
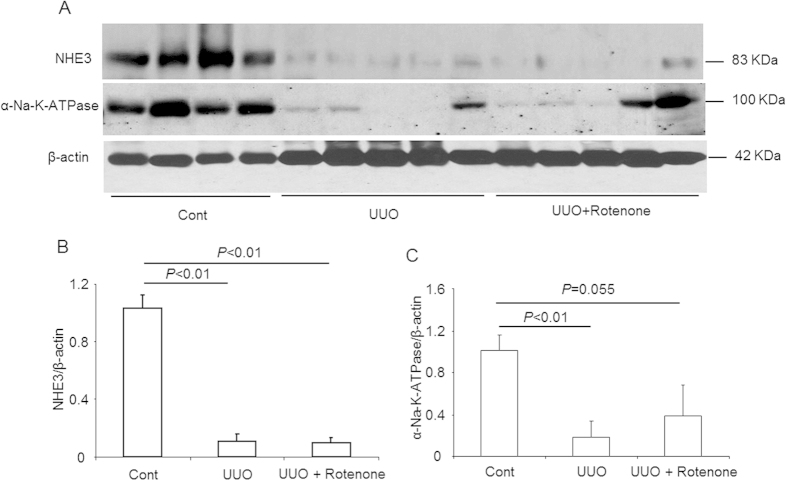
Protein expression of NHE3 and α-Na-K-ATPase in obstructed kidneys following rotenone treatment. (**A**) Western blot analysis of NHE3 and α-Na-K-ATPase. (**B**) Densitometric analysis of NHE3. (**C**) Densitometric analysis of α-Na-K-ATPase. The presented data are means ± SE. N = 4–5 in each group.

We further examined the protein levels of the collecting duct sodium channels ENaCα, ENaCβ, and ENaCγ. Similar to NKCC2, downregulation of ENaCα was reversed by rotenone treatment, as determined via immunohistochemistry and Western blotting (Fig. 3A–C). However, the ENaCβ protein was not affected by kidney obstruction or rotenone treatment (Fig. 4A,B). For ENaCγ, the non-cleaved form (85 kDa) was not reduced in obstructed kidneys (Fig. 5A,C). Interestingly, rotenone treatment caused greater induction of non-cleaved ENaCγ (Fig. 4A,C). In contrast, the cleaved form (70 kDa) of ENaCγ disappeared entirely in the obstructed kidneys, which was not affected by rotenone (Fig. 4A,D).

### Effects of mitochondrial complex-I inhibition on the protein expression of NHE3 and α-Na-K-ATPase in obstructed kidneys

Through Western blotting, we further examined the regulation of the NHE3 and α-Na-K-ATPase proteins. As shown in Fig. 5, both NHE3 and α-Na-K-ATPase were markedly decreased in obstructed kidneys, and rotenone administration had no significant effect on these changes.

### Effects of mitochondrial complex-I inhibition on the kidney content of PGE2 and Ang II and the mRNA expression of endothelin (ET)1 and angiotensinogen (AGT) in obstructed kidneys

**Figure 6:**
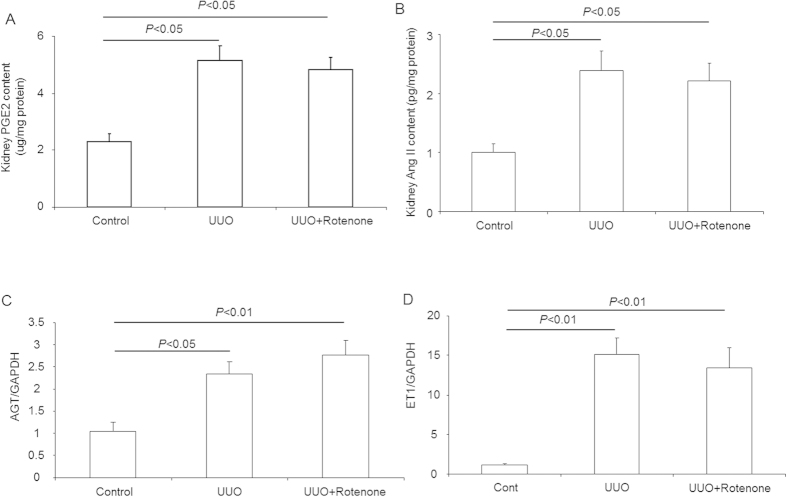
Kidney contents of PGE2 and Ang II and mRNA expression of AGT and ET1 in obstructed kidneys following rotenone treatment. (**A**) Kidney content of PGE2 determined via EIA. (**B**) Kidney content of Ang II determined via ELISA. (**C**) mRNA expression of AGT determined via qRT-PCR. (**D**) mRNA expression of ET1 determined via qRT-PCR. The presented data are means ± SE. N = 5 in each group.

A review of the literature revealed that both PGE2 and Ang II have been reported to play roles in mediating the reduction of sodium transporters in obstructive kidney disease. Therefore, we examine the contents of PGE2 and Ang II in obstructed kidneys with or without rotenone treatment. Surprisingly, the induction of PGE2 and Ang II was not altered by rotenone administration (Fig. 6A,B). In agreement with the unaltered Ang II content, the upregulation of AGT was not impacted by rotenone, as determined by qRT-PCR (Fig. 6C). Based on the known role of ET1 in the regulation of sodium transporters, we also examined the mRNA level of ET1 and observed robust stimulation of ET1 in obstructed kidneys, which was not affected by rotenone administration (Fig. 6D). These results suggested that PGE2, Ang II, and ET1 might not be attributable to the effects of rotenone on sodium transporter regulation in obstructed kidney.

### Effects of mitochondrial complex-I inhibition on atrial natriuretic peptide (ANP), brain natriuretic peptide (BNP), and C-type natriuretic peptide (CNP) expression in obstructed kidneys

**Figure 7:**
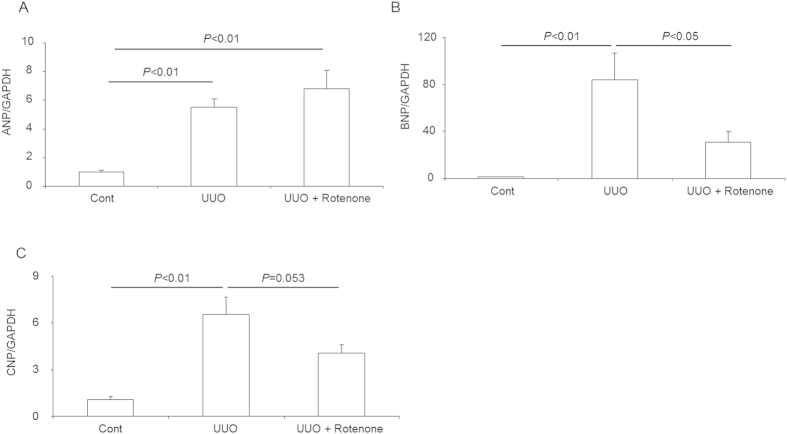
mRNA expression of natriuretic peptides in obstructed kidneys following rotenone treatment. (**A**) qRT-PCR analysis of ANP. (**B**) qRT-PCR analysis of BNP. (**C**) qRT-PCR analysis of CNP. The presented data are means ± SE. N = 5 in each group.

Renal natriuretic peptides exhibit an established role in regulating sodium transporters in the kidney. Here, we detected the mRNA expression of three forms of natriuretic peptides (ANP, BNP, and CNP). As expected, the three forms of natriuretic peptides were all markedly enhanced in the obstructed kidneys (Fig. 7A–C). Rotenone treatment resulted in significant blockade of BNP and a trend of CNP attenuation but showed no effect on ANP (Fig. 7A–C). These data indicated that suppression of BNP and/or CNP could be beneficial in preventing the downregulation of NKCC2 and ENaC in obstructive kidney disease.

### Effects of mitochondrial complex-I inhibition on nitric oxide synthases in obstructed kidneys

**Figure 8:**
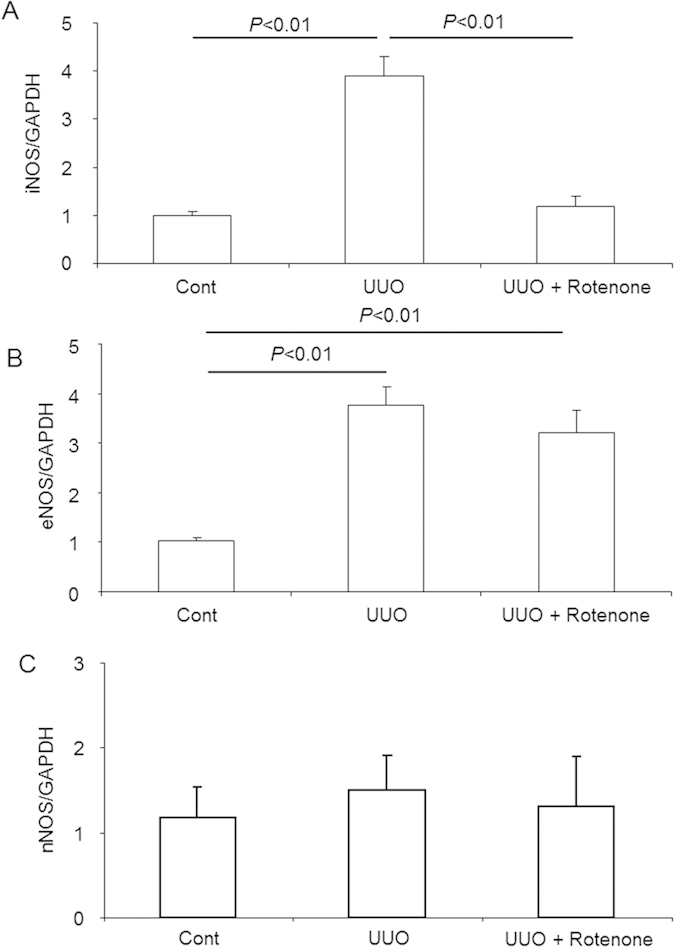
mRNA expression of nitric oxide synthases in obstructed kidneys following rotenone treatment. (**A**) qRT-PCR analysis of iNOS. (**B**) qRT-PCR analysis of eNOS. (**C**) qRT-PCR analysis of nNOS. The presented data are means ± SE. N = 5 in each group.

To further study the potential mechanisms involved in the effects of rotenone on regulating NKCC2 and ENaC, we examined nitric oxide synthases including iNOS, eNOS, and nNOS via qRT-PCR. As shown by the data, iNOS and eNOS, but not nNOS, were elevated in the obstructed kidneys (Fig. 8A–C). Following rotenone treatment, iNOS, but not eNOS, was normalized (Fig. 8A), suggesting a potential for iNOS-derived nitric oxide to contribute to the effect of rotenone on modulating sodium transporters in obstructed kidneys.

## Discussion

Obstructive kidney disease is a very common clinical complication. Kidney obstruction leads to a rapid downregulation of sodium transporters within 24 to 72 hours and time-dependent development of tubulointerstitial fibrosis^8^,^14^,^15^. The release of kidney obstruction is accompanied by substantial polyuria and salt waste^14^,^15^, which could result in fluid imbalance and electrolyte disorders. In the past several decades, a number of investigations related to the pathogenic mechanisms underlying the obstruction-induced downregulation of renal sodium transporters have been performed. Nørregaard R *et al.* demonstrated that COX-2/prostaglandin E2 signaling might be a mechanism for inducing the dysregulation of renal sodium transporters^9^. Later, this group further reported that Ang II is also involved in a pathogenic mechanism in this phenomenon^8^. These distinct findings indicated the complexity of the mechanisms associated with this pathological process and triggered our interest in further studying this phenomenon.

Mitochondrial injury has been observed in obstructive kidneys^11^,^12^,^13^. Mitochondrial injury causes ATP depletion, reactive oxygen species (ROS) overproduction, and the release of proapoptotic factors such as cytochrome C and mitochondrial DNA, which is involved in the tissue damage. Recently, we found that inhibition of mitochondrial complex-I attenuated renal fibrosis in obstructed kidneys^13^, in accord with the observed ameliorated mitochondrial dysfunction and ROS production^13^. In the present study, we further investigated the role of the mitochondria in regulating renal sodium transporters in obstructive nephropathy via the administration of rotenone, an established inhibitor of mitochondrial complex-1. As expected, the mRNA levels of most sodium transporters, including NHE3, NCC, NKCC2, α-Na-K-ATPase, and ENaCα, were markedly downregulated, as determined via qRT-PCR. The mRNA expression of ENaCβ and ENaCγ was not affected by kidney obstruction. Following the administration of rotenone, the reduction of the mRNA levels of NKCC2 and ENaCα, but not the other sodium transporters, was significantly attenuated. Through Western blotting, we found that the downregulation of NKCC2, p-NKCC2, and ENaCα was entirely reversed by rotenone. However, the protein levels of other transporters, including NHE3, NCC, α-Na-K-ATPase, ENaCβ, and ENaCγ, were not altered by rotenone. Recent studies from our and other groups demonstrated that rotenone treatment attenuated oxidative response in obstructive kidneys^13^,^16^. To define the role of mitochondrial oxidative stress in regulating sodium transporters in obstructive kidney disease needs future experimental evidence. Moreover, the mechanism for rotenone effect on ENaCα regulation, but not other ENaC subunits in this experimental setting is unclear. However, the distinct regulation of ENaC subunits in other experimental settings was also reported previously^17^,^18^. These results indicated a selective role of rotenone in regulating renal sodium transporters in the context of chronic kidney obstruction.

We further examined the kidney contents of PGE2 and Ang II and found that both were markedly elevated by kidney obstruction. However, neither was affected by rotenone. Meanwhile, the upregulation of angiotensinogen mRNA expression was not inhibited by rotenone. These data suggested that the effects rotenone on protecting NKCC2 and ENaCα downregulation are independent of PGE2 and Ang II. Due to the known role of ET1 in modulating renal sodium transporters^19^,^20^,^21^, we detected the mRNA levels of ET1 in the kidney. Similar to PGE2 and Ang II, the upregulation of renal ET1 was unchanged in animals subjected to rotenone treatment, which suggested that ET1 may not play a role in mediating the effects of rotenone during this process.

Natriuretic peptides, including atrial natriuretic peptide (ANP), brain natriuretic peptide (BNP), and C-type natriuretic peptide (CNP), are expressed in the kidneys and play important roles in regulating renal sodium transporters^22^,^23^,^24^,^25^,^26^. Therefore, we examined the regulation of these natriuretic peptides via qRT-PCR. Strikingly, all of the peptides were upregulated in obstructed kidneys, and rotenone treatment significantly reduced BNP level and resulted in a trend of CNP suppression. The enhancement of ANP was unchanged in rotenone-treated animals. These data suggested that BNP might be attributable to the effects of rotenone on regulating NKCC2 and ENaCα in obstructed kidneys. By now, there is no direct evidence indicating the relationship between mitochondrial function and natriuretic peptides. However, inflammation has been reported to be associated with the enhancement of plasma BNP and CNP^27^,^28^. Considering the anti-inflammatory effect of rotenone in obstructive nephropathy^13^, we could hypothesize that rotenone might attenuate the increments of BNP and CNP via an anti-inflammatory mechanism. Moreover, a study to evaluate the role of natriuretic peptides in this pathological process via inhibiting the activity of BNP and/or CNP is still required in the future.

Another known regulator of renal sodium transporters is nitric oxide (NO)^29^,^30^,^31^. NO is generated by three nitric oxide synthases (iNOS, eNOS, and nNOS). Through qRT-PCR analysis, we observed robust upregulation of iNOS and eNOS, but not nNOS, in obstructed kidneys. Importantly, rotenone selectively and completely normalized iNOS induction. These interesting findings suggested that inhibition of iNOS-derived NO by rotenone might contribute to the effects of rotenone in this experimental setting to some extent. Both inflammation and oxidative stress contribute to iNOS induction^32^,^33^, thus rotenone abolished iNOS induction might through its activities of anti-inflammation and anti-oxidative stress^13^,^16^.

In summary, employing the mitochondrial complex-1 inhibitor rotenone, we observed an interesting phenomenon, in which inhibition of mitochondrial complex-1 selectively reversed the obstruction-induced downregulation of NKCC2 and ENaCα in kidneys. The distinct responses of sodium transporters to rotenone treatment indicated that the downregulation of NKCC2 and ENaC might be associated with a different pathogenic mechanism than observed for other sodium transporters in obstructive nephropathy. The role of the mitochondria may be particularly important for the reduction of NKCC2 and ENaCα, but not the other transporter. These findings not only shed new light on our understanding of the mechanisms underlying the obstruction-related dysregulation of renal sodium transporters, but also suggest new targets for addressing renal concentrating defects in obstructive nephropathy.

## Methods

### Animals

C57BL/6J mice were originally purchased from the Jackson laboratory. This mouse colony was propagated at Nanjing Medical University. In all experiments, 3- to 4-mo-old male mice were used. All of the mice were maintained under a 12:12-h light-dark cycle (lights on at 6:00 a.m. and lights off at 6:00 p.m.). Animal studies were performed under protocols in accordance with relevant guidelines and regulations and approved by the Nanjing Medical University Institutional Animal Care and Use Committee.

### Establishment of the UUO mouse model

Unilateral ureter obstruction was induced as previously described^13^,^34^. Briefly, the left ureter was exposed and subsequently ligated with 6.0 silk through a small abdominal incision under anesthesia with 2.0% isoflurane. The abdomen was closed in two layers. All of the mice received analgesia (subcutaneous injection of 50 μg/kg buprenorphin (Temgesic, Shering-Plough) after the surgery. Following the surgery, a jelly diet with or without rotenone at a dose of 500 ppm was given to the UUO mice. The sham control mice were treated with the jelly diet without rotenone. After seven days of UUO and rotenone treatment, the mice (N = 5 per group) were sacrificed, and their kidney tissue was harvested for the analysis of gene and protein expression and histological changes.

### Immunohistochemistry

The kidneys were fixed with 10% formalin and embedded in paraffin. Kidney sections (4-μm thickness) were incubated in 3% H_2_O_2_ for 15 minutes at room temperature to block endogenous peroxidase activity. After boiling in antigen retrieval solution (1 mmol/L Tris-HCl, 0.1 mmol/L EDTA, pH = 8.0) for 15 minutes at high power in a microwave oven, the sections were incubated overnight at 4 °C with a rabbit anti-NKCC2 antibody (Stressmarq Biosciences Inc., Canada) or a rabbit anti-ENaCα antibody (Stressmarq Biosciences Inc., Canada). After washing with PBS, the secondary antibody was applied, and the signal was visualized using an ABC kit (Santa Cruz Biotechnology).

### Immunoblotting

The whole kidney was lysed, and the protein concentration was determined using the Coomassie reagent. Protein (60 μg) from whole kidney lysates was denatured in boiling water for 10 min, separated via SDS-polyacrylamide gel electrophoresis, and transferred to nitrocellulose membranes. The blots were blocked overnight with 5% nonfat dry milk in Tris-buffered saline (TBS), followed by incubation for 1 h with rabbit anti-NHE3 (Abcam, Cambridge, MA), anti-α-Na-K-ATPase (Abcam), anti-NKCC2 (Stressmarq Biosciences Inc., Canada), anti-ENaCα (Stressmarq Biosciences Inc., Canada), anti-ENaCβ (Stressmarq Biosciences Inc., Canada) or anti-ENaCγ (Stressmarq Biosciences Inc., Canada) at a dilution of 1:1,000 After being washed with TBS, blots were incubated with a goat anti-horseradish peroxidase-conjugated secondary antibody (1:1,000 dilution) and visualized using ECL kits (Amersham, Piscataway, NJ USA).

### qRT-PCR

Total RNA isolation and reverse transcription were performed as previously described^35^. mRNA was determined via qRT-PCR. Oligonucleotides were designed using Primer3 software (available at http://frodo.wi.mit.edu/primer3/), and the sequences are shown in Table 1. qRT-PCR amplification was performed using SYBR Green Master Mix (Applied Biosystems, Warrington, UK) and the Prism 7500 Real-Time PCR Detection System (Applied Biosystems, Foster City, CA, USA). The cycling conditions were 95 °C for 10 min, followed by 40 cycles of 95 °C for 15 s and 60 °C for 1 min.[Table t1]

### Enzyme Immunoassay (EIA)

Kidney tissue was homogenized in phosphate-buffered saline, followed by centrifugation for 5 min at 10,000 r.p.m. The supernatant was diluted 1:50 with enzyme immunoassay buffer. The concentration of PGE2 was determined using an enzyme immunoassay according to the manufacturer’s instructions (Cayman, Ann Arbor, MI). The kidney content of An II was measured with EIA kits (Cayman, Ann Arbor, MI).

### Statistical Analysis

All values are presented as the mean ± SE. Statistical analysis was performed using ANOVA followed by multiple comparison test. Differences were considered to be significant when *P* < 0.05.

## Additional Information

**How to cite this article**: Zhang, Y. *et al.* Inhibition of Mitochondrial Complex-1 Prevents the Downregulation of NKCC2 and ENaCa in Obstructive Kidney Disease. *Sci. Rep.* **5**, 12480; doi: 10.1038/srep12480 (2015).

## Figures and Tables

**Table 1 t1:** Sequences of Primers for real-time PCR.

**Gene**	**Primer sequence**	**Accession Number**
GAPDH	5′-gtcttcactaccatggagaagg-3′	M32599
	5′-tcatggatgaccttggccag-3′	
EnaCα	5′-gcttcatctttacctgtcgtttc-3′	NM_011324
	5′-ccagagattggagttgttcttgt-3′	
ENaCβ	5′-cagtggggagtcttcatcc-3′	NM_011325
	5′-tcctggtggtgttgctgt-3′	
EnaCγ	5′-ctgcttcttcgatgggatg-3′	NM_011326
	5′-gacaccaggaaggggttgt-3′	
NCC	5′-gacaggcaccaacagtgaga-3′	U61085
	5′-tagagatggcggagatggag-3′	
NKCC2	5′-gctcttcattcgcctctcct-3′	NM_011389
	5′-agcctattgacccaccgaac-3′	
NHE3	5′-ctgaggaggaaccgagca-3′	XM_993032
	5′-aggcccagaacgatgagtag-3′	
Α-Na-K-ATPase	5′-tgctctcttctctttctagtctcc-3′	NM_144900
	5′-gctcatccatatccctttcc -3′	
Nnos	5′-cagccaaagcagagatgaaa-3′	D14552
	5′-atacgggttgttgaggacca-3′	
iNOS	5′-actgtgtgcctggaggttct-3′	NM_010927
	5′-tctctgcctatccgtctcgt-3′	
eNOS	5′-gagagcgagctggtgtttg-3′	NM_008713
	5′-tgatggctgaacgaagattg-3′	
ANP	5′-ccgatagatctgccctcttg-3′	NM_008725
	5′-atcgactgccttttcctcct-3′	
BNP	5′-cctcacaaaagaacacccaaa-3′	NM_008726
	5′-ggaaagagacccaggcaga-3′	
CNP	5′-tacaaaggcggcaacaaga-3′	NM_010933
	5′-agatgctggaggctgatgac-3′	
AGT	5′-tgtgacagggtggaagatga-3′	NM_007428
	5′-agatcatgggcacagacacc-3′	
ET1	5′-cgctgttcctgttcttcctt-3′	NM_010104
	5′-ctggtctgtggccttattgg-3′	
